# Chelation of Theranostic Copper Radioisotopes with S-Rich Macrocycles: From Radiolabelling of Copper-64 to In Vivo Investigation

**DOI:** 10.3390/molecules27134158

**Published:** 2022-06-28

**Authors:** Marianna Tosato, Marco Verona, Chiara Favaretto, Marco Pometti, Giordano Zanoni, Fabrizio Scopelliti, Francesco Paolo Cammarata, Luca Morselli, Zeynep Talip, Nicholas P. van der Meulen, Valerio Di Marco, Mattia Asti

**Affiliations:** 1 Department of Chemical Sciences, University of Padova, Via Marzolo 1, 35131 Padova, Italy; marianna.tosato@unipd.it (M.T.); giordano.zanoni@unipd.it (G.Z.); valerio.dimarco@unipd.it (V.D.M.); 2 Department of Pharmaceutical Sciences, University of Padova, Via Marzolo 8, 35131 Padova, Italy; marco.verona@phd.unipd.it; 3 Center for Radiopharmaceutical Sciences, Paul Scherrer Institute, 5232 Villigen, Switzerland; chiara.favaretto@psi.ch (C.F.); zeynep.talip@psi.ch (Z.T.); nick.vandermeulen@psi.ch (N.P.v.d.M.); 4 Nuclear Medicine Department, Cannizzaro Hospital, Via Messina 829, 95126 Catania, Italy; marco.pometti@gmail.com (M.P.); fabrizioscopelliti@gmail.com (F.S.); 5 Institute of Molecular Bioimaging and Physiology, National Research Council (IBFM-CNR), 90015 Cefalù, Italy; francesco.cammarata@ibfm.cnr.it; 6 Legnaro National Laboratories, Italian Institute of Nuclear Physics, Viale Dell’Università 2, 35020 Padova, Italy; luca.morselli@lnl.infn.it; 7 Laboratory of Radiochemistry, Paul Scherrer Institute, 5232 Villigen, Switzerland; 8 Radiopharmaceutical Chemistry Section, Nuclear Medicine Unit, AUSL-IRCCS di Reggio Emilia, Via Amendola 2, 42122 Reggio Emilia, Italy

**Keywords:** copper-64, chelators, radiopharmaceuticals, cancer

## Abstract

Copper radioisotopes are generally employed for cancer imaging and therapy when firmly coordinated via a chelating agent coupled to a tumor-seeking vector. However, the biologically triggered Cu^2+^-Cu^+^ redox switching may constrain the in vivo integrity of the resulting complex, leading to demetallation processes. This unsought pathway is expected to be hindered by chelators bearing N, O, and S donors which appropriately complements the borderline-hard and soft nature of Cu^2+^ and Cu^+^. In this work, the labelling performances of a series of S-rich polyazamacrocyclic chelators with [^64^Cu]Cu^2+^ and the stability of the [^64^Cu]Cu-complexes thereof were evaluated. Among the chelators considered, the best results were obtained with 1,7-bis [2-(methylsulfanyl)ethyl]-4,10,diacetic acid-1,4,7,10-tetraazacyclododecane (DO2A2S). DO2A2S was labelled at high molar activities in mild reaction conditions, and its [^64^Cu]Cu^2+^ complex showed excellent integrity in human serum over 24 h. Biodistribution studies in BALB/c nude mice performed with [^64^Cu][Cu(DO2A2S)] revealed a behavior similar to other [^64^Cu]Cu-labelled cyclen derivatives characterized by high liver and kidney uptake, which could either be ascribed to transchelation phenomena or metabolic processing of the intact complex.

## 1. Introduction

The last few decades have witnessed a revolution in the understanding of the intersection between the coordination chemistry of metallic radionuclides and nuclear medicine applications, opening new frontiers for cancer imaging and therapy. The majority of the situations have seen the success of these approaches being stringently dependent upon the firm coordination of a α, *β*^−^, Auger electrons or γ-ray-emitting radiometal via a chelator covalently tethered to a tumor-targeting vector [1,2,3,4]. Indeed, any radiometal release from the chelate in a biological environment, due to possible proton-assisted or transchelation/transmetallation pathways, represents a particular concern for clinical applications owing to the high background noise and the undesirable dose to off-target organs [1,4,5,6,7,8]. This undesired phenomenon can be hampered if the chelating agent is specifically tailored for the coordination chemistry of the radiometal and it is able to form a highly thermodynamically stable and kinetically inert complex in vivo. Rapid chelation under mild reaction conditions is also an essential feature for the radiolabelling of thermally- and pH-sensitive targeting agents (e.g., antibodies) that are vulnerable to unfolding or degradation under extreme conditions [2,3,9].

Among the variety of medically relevant transition metals, copper draws great interest from the nuclear medicine community as it possesses an attractive combination of isotopes ideal for both diagnostic and therapeutic, i.e., theranostic, applications. The theranostic approach represents a very promising strategy able to predict whether a patient will benefit from therapy, thereby avoiding unnecessary or ineffective treatments [10,11]. In particular, copper-64 (*t*_1/2_ = 12.7 h) possesses a rare emission profile which combines *β*^+^ (*E_β_*^+^_, max_ = 655 keV, *I_β_*^+^ = 18%), *β*^−^ (*E_β_*^−^_, max_ = 573 keV, *I_β_*^−^ = 39%,) and electron capture emissions (*I*_EC_ = 43%). The relatively low *β*^+^-energy is comparable to that of fluorine-18, and the resulting annihilation photons produce high-quality images [11,12,13,14,15,16]. Moreover, its relatively long half-life can enable positron emission tomography (PET) imaging at later time points than for fluorine-18. Although its decay profile would make copper-64 a dual diagnostic and therapeutic radionuclide, the high positron branching ratio precludes the latter use [11]. However, copper-64 retains the potential for theranostic applications when paired with the pure *β*^−^ emitter copper-67 (*t*_1/2_ = 61.9 h, *E_β_*^−^_, max_ = 141 keV, *I_β_*^−^ = 100%) [12,13,14,15].

**Figure 1:**
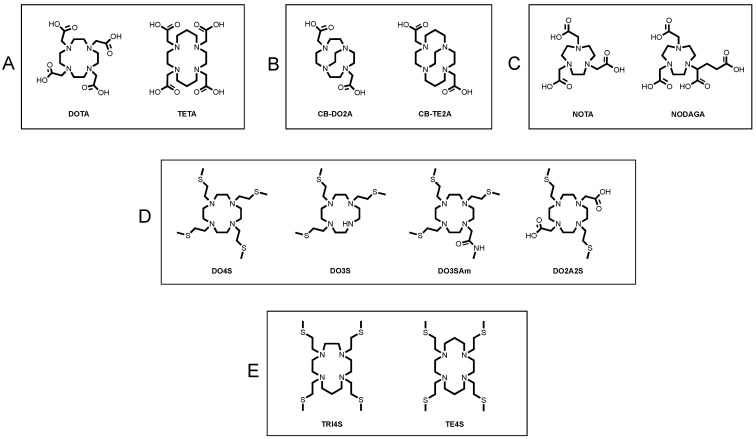
Structure of (**A**–**C**) state-of-the-art ligands for [^64^Cu]Cu^2+^ chelation, (**D**) cyclen-based sulfur-containing ligands and (**E**) non-cyclen-based sulfur-containing ligands.

Cu^2+^ being a hard-borderline Lewis acid with a predilection for hard-borderline donors, polyazamacrocyclic-based ligands have dominated its in vivo chelation so far. Polyamminocarboxylic macrocycles such as 1,4,7,10-tetraazacyclododecane-1,4,7,10-tetraacetic acid (DOTA), 1,4,8,11-tetraazacyclotetradecane-1,4,8,11-tetraacetic acid (TETA) and their derivatives (Figure 1A) provided excellent binding environments for Cu^2+^, as reflected by the high thermodynamic stability of the resulting cupric complexes. However, they have not shown sufficient inertness in vivo, leading to an accumulation of unbound radiometal in liver and kidneys of murine models [5,8,15,16,17,18,19]. To improve the biological stability of the [^64^Cu]Cu-complexes, a large variety of modifications of the azamacrocyclic backbone have been performed. Chelators based on cross-bridged platforms (e.g., 1,4,7,10-tetraazabicyclo[5.5.2]tetradecane-4,10-diacetic acid—CB-DO2A or 1,4,8,11-tetraazabicyclo[6.6.2]hexadecane-4,11-diacetic acid—CB-TE2A) possess high kinetic inertness but necessitate harsh labelling conditions, which limit their use to non-thermally sensitive biological vectors (Figure 1B) [17,20,21,22,23]. Hexa-azacages (e.g., DiamSar), bispiridine (e.g., 3,7-diazabicyclo[3.3.1]nonane), 1,4,7-triazacyclononane-1,4,7-triacetic acid (NOTA) and its derivatives (e.g., 1,4,7-triazacyclononane,1-glutaric acid-4,7-acetic acid—NODAGA) have also demonstrated the ability to form [^64^Cu]Cu-complexes with remarkable integrity (Figure 1C) [24,25,26]. Despite these considerations, the concern over the in vivo stability still elicits the development of improved alternatives to the current state-of-the-art [^64/67^Cu]Cu-chelating agents.

In particular, a potential dissociation pathway leading to in vivo demetallation is the biologically-triggered redox switching between Cu^2+^ and Cu^+^. This event can likely cause the premature release of copper radionuclides from the complex due to the inability of the borderline/hard-donors-containing chelator to retain the reduced copper form [1,27,28,29]. This redox-mediated dissociation mechanism can be mitigated either by preventing the bioreduction of the cupric center or by improving the coordination of the generated cuprous ions [1,30,31].

The use of chelators able to stabilize both copper oxidation states has been rarely considered so far, but it could be the key to obtain highly stable [^64/67^Cu]Cu-labelled radiotracers able to afford more effective and safer diagnostic and therapeutic protocols. In view of the acknowledged preference of Cu^+^ for soft donor groups, we have recently designed and developed a series of *N*-functionalized polyazamacrocycles bearing sulfur-containing pendant arms (Figure 1D,E) with the aim to stabilize the Cu^+^-oxidation state as well.

In the first series of ligands, a progressively increased number of thioether chains were appended to the 1,4,7,10-tetrazacyclodedecane (cyclen) backbone to modulate their chemical softness character [32]. 1,4,7,10-Tetrakis[2-(methylsulfanyl)ethyl]-1,4,7,10-tetraazacyclododecane (DO4S) possess four sulfanyl pendants while 1,4,7-tris[2-(methylsulfanyl)ethyl]-1,4,7,10-tetraazacyclododecane (DO3S) and its amido derivative, 1,4,7-tris[2-(methylsulfanyl)ethyl]-10-acetamido-1,4,7,10-tetraazacyclododecane (DO3SAm), hold three sulfur donors. In the latter, an amide chain was introduced to simulate the electronic and steric effect of an *N*-alkylation of DO3S when conjugated to a tumor-targeting molecule. Finally, in 1,7-bis[2-(methylsulfanyl)ethyl]-4,10,diacetic acid-1,4,7,10-tetraazacyclododecane (DO2A2S), the two opposite carboxylic arms of DOTA have been replaced with sulfur-containing ones, to produce a chelator with hybrid coordination capability between DOTA and DO4S (Figure 1D) [32]. In the second series, the effect of the ring size on the properties of the resulting copper complexes was considered, leading to 1,4,7,10-tetrakis[2-(methylsulfanyl)ethyl]-1,4,7,10-tetrazacyclotridecane (TRI4S) and 1,4,8,11-tetrakis[2-(methylsulfanyl)ethyl]-1,4,8,11-tetrazacyclotetradecane (TE4S) (Figure 1E) [33]. As a result, the thermodynamic, kinetic and structural properties of the Cu^2+/+^ complexes formed with these sulfur-bearing ligands were investigated demonstrating that they can be promising scaffolds for cutting-edge copper-based radiopharmaceuticals [1,33].

The encouraging results prompted us to further evaluate our chelators in a radioactive environment to fully assess their candidacy for nuclear medicine applications. Herein, the performances of the aforementioned series of sulfur-rich polyazamacrocycles towards the labelling of [^64^Cu]Cu^2+^ are detailed. Moreover, the stability in sundry competing conditions and in physiological media of the resulting complexes, as well as the in vivo biodistribution of the most promising candidate, are evaluated.

## 2. Results and Discussion

### 2.1. Copper-64 Radiolabelling

Radiolabelling experiments were performed under different reaction conditions of temperature and pH, across six orders of magnitude of chelator concentration to assess the capacity of sulfur-bearing ligands to chelate [^64^Cu]Cu^2+^. For comparison purposes, parallel experiments were also conducted using a radiotracer endowed with NODAGA as chelation moiety (namely NODAGA-RGD). We are acquainted that the presence of a targeting vector linked to the chelation moiety usually determines negative effects on the labelling performance of the chelator itself, however, NODAGA is a ligand able to bind copper efficiently and NODAGA-RGD was chosen because of its previously-attested capability of labelling the cyclotron-produced copper-64 at high molar activity [34].

The radiochemical incorporations (RCIs) were determined using both radio-UHPLC and radio-TLC. Representative radio-chromatograms are reported in Figures S1 and S2.

#### 2.1.1. Copper-64 Radiolabelling with Cyclen-Based S-Rich Chelators

**Figure 2:**
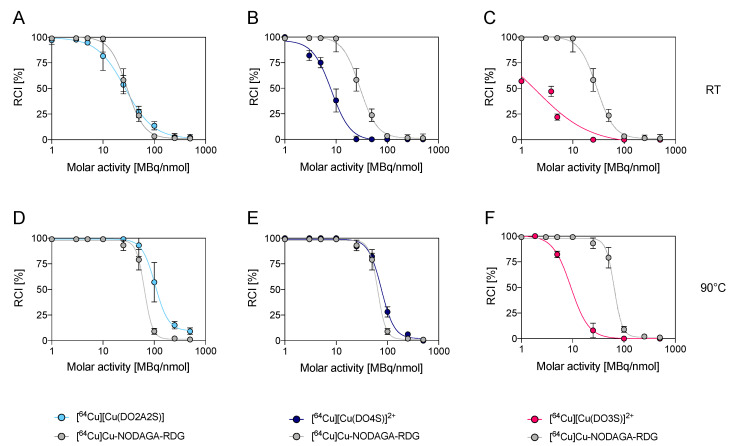
[^64^Cu]Cu^2+^ incorporation yields (10 min, pH 4.5) at different molar activities for DO2A2S, DO4S and DO3S. (**A**–**C**): RT (**D**–**F**): 90 °C. Incorporations are compared with those obtained with NODAGA-RDG under the same conditions.

**Figure 3:**
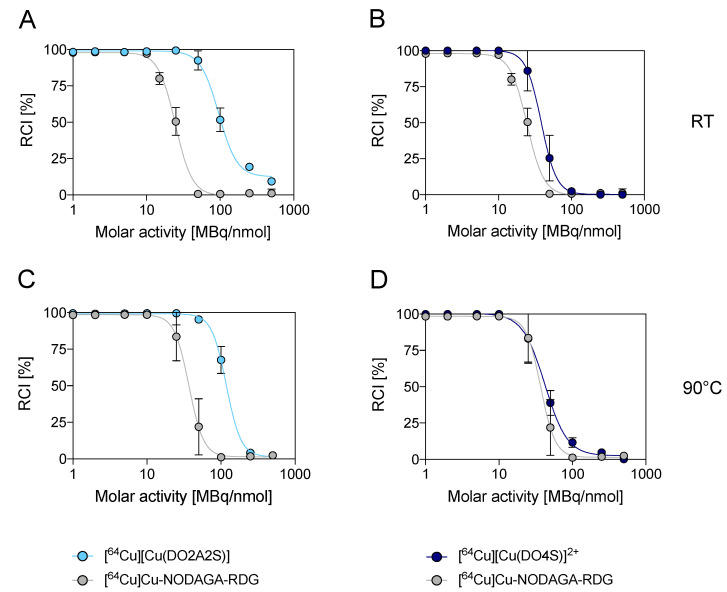
[^64^Cu]Cu^2+^ incorporation yields (10 min, pH 7) at different molar activities for DO2A2S and DO4S. (**A**,**B**): RT; (**C**,**D**): 90 °C. Incorporations are compared with those obtained with NODAGA-RDG under the same conditions.

The results obtained in the radiolabelling tests of DO2A2S, DO4S, and DO3S are illustrated in Figure 2 and Figure 3 and summarized in Tables S1 and S2. At RT and mild-acidic pH conditions (pH 4.5), NODAGA-RGD was able to quantitatively (>99%) chelate [^64^Cu]Cu^2+^ at a molar activity up to 10 MBq/nmol in 10 min while, at this molar activity, DO2A2S, DO4S, and DO3S yielded ~82%, 40% and 20%, respectively. To obtain the same quantitative incorporation, DO2A2S needed a halved [^64^Cu]Cu^2+^ concentration (5 MBq/nmol) whilst for DO4S and DO3S a 10-fold and 1000-fold lower molar activity was required. Incorporation yields of NODAGA-RGD and DO2A2A also followed superimposable trends both being ~58% at 25 MBq/nmol and decreasing below 10% at molar activity >100 MBq/nmol. Conversely, no radiometal incorporation was observed for both DO4S and DO3S at molar activities >25 MBq/nmol (Figure 2A–C). A more inferior labelling behavior was obtained with DO3SAm, which was not able to complex [^64^Cu]Cu^2+^, even at the highest ligand concentration used (2 × 10^−3^ M corresponding to 0.01 MBq/nmol).

The severely reduced efficiency of DO4S, DO3S and DO3SAm with respect to the carboxylate-containing chelators (DO2A2S and NODAGA-RDG) can be attributed not only to a lower thermodynamic stability of their Cu^2+^ complexes, but also to the slower kinetics of the complexation reaction owing to the non-anionic nature of the sulfur pendant arms, as previously pointed out with stable Cu^2+^ complexes [1]. At pH 4.5, the negatively-charged carboxylic arms can interact with the metal ion and rapidly form an out-of-cage intermediate later converted in an in-cage product. While this process is feasible for NODAGA and DO2A2S, it is absent when all the carboxylic arms are amidated or replaced with sulfur-containing pendants such as in DO4S, DO3S, or DO3SAm. Conversely, the removal of only two carboxylic arms in DO2A2S led to a less marked, but meaningful, decrease in the labelling performance—likely only due to probabilistic reasons. As shown in Figure S3, prolonged reaction times had beneficial effects on the incorporation obtained with DO4S and DO3S, thus, corroborating the hypothesis that their labelling efficiencies are limited by kinetic barriers. As shown in Figure 2D,E, [^64^Cu]Cu^2+^ incorporation of DO2A2S and DO4S was strongly enhanced at 10 min reaction time, when the temperature was increased to 90 °C. In this condition, DO2A2S was able to almost quantitatively incorporate the radiometal up to 50 MBq/nmol, also exceeding the performances of NODAGA-RGD (<80% incorporation therein). On the other hand, DO4S was able to label at a maximum molar activity equal to 10 MBq/nmol. NODAGA-RGD was influenced by high temperature as well, although to a lesser extent than DO2A2S and DO4S. In fact, its quantitative incorporation increased only from 10 MBq/nmol to 25 MBq/nmol. At 90 °C, DO3S (Figure 2F) and DO3SAm efficiencies were also improved and were able to afford quantitative labelling at 2 MBq/nmol. However, these results are still very low if compared to those obtained for the other chelators.

When the labelling experiments were performed at RT and in neutral conditions (pH 7), an outstanding reduction of the maximum ligand concentration needed to obtain quantitative radiometal incorporation was found for DO2A2S and DO4S. On the other hand, the effects on the complexation with NODAGA-RGD were almost negligible (Figure 3A,B). As a result, under these conditions, DO2A2S strongly outperformed NODAGA-RGD, attaining quantitative incorporation up to 50 MBq/nmol after 10 min and exhibiting a 52 ± 8% and 9 ± 2% incorporation from 100 MBq/nmol to 500 MBq/nmol. DO4S still had inferior performance compared to DO2A2S but achieved a labelling efficacy comparable, if not superior to, NODAGA-RGD. The molar activity reached by DO4S for the quantitative incorporation was 10 MBq/nmol (roughly the same obtained by NODAGA-RGD) but, when the ligand concentration was reduced to give a molar activity equal to 25 MBq/nmol, the labelling efficiency decreased to 86 ± 14% for DO4S and ~50% for NODAGA-RGD, respectively. Due to the low performances found in the precedent tests, DO3S and DO3SAm behavior were not deeply investigated but they both showed a labelling efficiency >90% at 1 MBq/nmol.

The general improvement to the coordination properties shown at neutral pH by all the cyclen-based S-rich chelators can be likely ascribed to the presence of less protonated forms of the ligands which lowered the electrostatic repulsion towards [^64^Cu]Cu^2+^ ions, as emphasized by our previously-reported thermodynamic and kinetic data [1,32]. Conversely, for NODAGA-RDG, the pH variation has a negligible effect on the maximum molar activity, likely because the ligand possesses negatively-charged carboxylic arm(s) both at pH 4.5 and 7 [35].

As shown in Figure 3C,D, contrary to what occurred at pH 4.5, at neutral pH, the influence of the temperature on [^64^Cu]Cu^2+^ incorporation was minimal for all the chelators examined.

#### 2.1.2. Copper-64 Radiolabelling with Non-Cyclen-Based S-Rich Chelators

**Figure 4:**
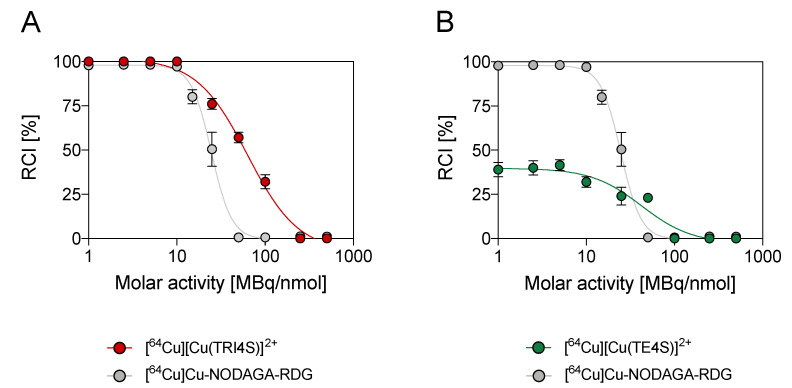
[^64^Cu]Cu^2+^ incorporation yields (10 min, pH 7 and RT) at different molar activities for (**A**) TRI4S and (**B**) TE4S. Incorporations are compared with those obtained with NODAGA-RDG under the same conditions.

Amongst the several labelling conditions screened for the cyclen-based ligands, the neutral pH medium and the ambient temperature proved to be the most effective. Hence, the consequence of increasing the azamacrocyclic ring size on the incorporation of [^64^Cu]Cu^2+^ was examined for two non-cyclen-based S-rich chelators only in these conditions. As reported in Figure 4A and Table S3, TRI4S (i.e., the 13-member ring analogue of DO4S) was able to quantitatively incorporate [^64^Cu]Cu^2+^ up to 10 MBq/nmol in less than 10 min while, at 25 and 500 MBq/nmol, the incorporation dropped to ~75% and 0%, respectively. These results are comparable to those obtained with DO4S and point out that the addition of a single carbon atom in the ligand scaffold does not markedly affect the labelling performances. This result agrees with the reported thermodynamic and kinetic data [33]. Conversely, TE4S (i.e., the 14-member ring ligand) showed markedly inferior labelling performance than DO4S and TRI4S as it always yielded modest results (Figure 4B and Table S3), with only ~40% incorporation achieved using the highest considered chelator concentration. The superior radiolabelling capacity of TRI4S over TE4S can be justified by the greater thermodynamic stability of its cupric complex [33]. [^64^Cu]Cu^2+^ complexation with TE4S can be enhanced by increasing the reaction temperature: the highest incorporation (e.g., from 24% at RT to 74% at 70 °C at 25 MBq/nmol) was however accompanied by the formation of labelling by-products. The inability of TE4S to achieve quantitative [^64^Cu]Cu^2+^ complexation precluded any further evaluation of this chelator.

### 2.2. Competition Assays

Competitive assays were performed using DOTA as a challenging agent for the cyclen-based chelators, as well as some metal cations (i.e., Zn^2+^ and Ni^2+^) that could outcompete with [^64^Cu]Cu^2+^ for the ligand binding. DOTA was chosen, as it was considered the perfect challenging agent to directly assess the influence of the S-containing pendant arms on the complexation ability of the cyclen-based chelators, while the aforementioned metal ions were selected as they are the decay products of copper-64 and, as a result, the most common metallic impurities in [^64^Cu]Cu^2+^ solutions. Moreover, Zn^2+^ also represents a biologically relevant cation and can be found at unsubtle concentrations in biological fluids [36].

**Figure 5:**
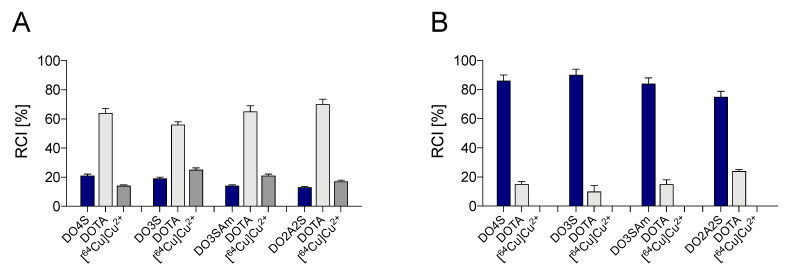
Relative abundance of the different [^64^Cu]Cu^2+^ complexes obtained with the sulfur-rich cyclen-based chelators in the challenge assays with DOTA (1:1 DOTA-to-chelator molar ratio) at (**A**) pH 4.5 and (**B**) pH 7 after 10 min reaction at RT.

In regard to the DOTA-competition, [^64^Cu][Cu(DOTA)]^2−^ complex was always the predominant compound formed at pH 4.5 after 10 min of reaction. However, the result was overturned at pH 7, where the complex formed with each sulfur-containing ligand prevailed in all cases. The results are reported in Figure 5, while representative radio-chromatograms are shown in Figures S4 and S5. This pH-dependent behavior can be rationalized considering the interplay between the kinetic and thermodynamic aspects that guide the Cu^2+^ complex formation. At low pH, the slowed-down reactivity of the sulfur-bearing ligands should become the leading factor. Contrarily, at neutral pH, the high thermodynamic stability of the S-rich cyclen-based macrocycles likely drives the formation of the complexes thereof as, according to our previously-reported data, the complex formation occurred with a rate comparable to DOTA [1].

In metal competition assays, an almost complete [^64^Cu]Cu^2+^ incorporation (always >90%) was achieved when the sulfur-containing ligands were mixed with the radiometal in the presence of a molar excess (2-fold with respect to the chelator of Zn^2+^ or Ni^2+^—Table 1). The results obtained pointed out a high selectivity of these ligands for Cu^2+^ with respect to other divalent cations (the molar ratio among cations and copper-64 exceeded 5000:1) which, in turn, may be related to the higher affinity of Cu^2+^ towards sulfur. An exception to this behavior is represented by DO2A2S, which was somewhat affected by the challenging environment (Table 1). In this case, the interaction with the competitive cations might have been favored by the presence of the carboxylic groups.

A further competitive assay was performed by adding an equimolar mixture of three cyclen-based sulfur-containing ligands, i.e., DO4S, DO3S, and DO3SAm, to [^64^Cu]Cu^2+^ to directly compare their labelling efficiency. The reaction mixture was incubated at room temperature and analyzed at different time points to observe which complex is produced first (“kinetic product”) and whether changes can be detected over time (“thermodynamic product”). The corresponding radio-chromatograms are shown in Figure S6 and the results reported in Figure S7. DO2A2S was confirmed to be the most efficient ligand among the series as it incorporated more than 80% of [^64^Cu]Cu^2+^ after only a few minutes of reaction and kept this incorporation constant over time. This is likely due to the synergy between the stronger thermodynamic stability and the faster formation kinetic of its cupric complex [1].

### 2.3. Stability Assays with Challenging Agents

Stability assays were conducted to predict the in vivo integrity of the [^64^Cu]Cu^2+^ complexes with the ligands investigated. Experiments were executed by incubating the preformed [^64^Cu]Cu^2+^ complexes in progressively more challenging media. The concentrations of challenging agents utilized in these experiments are not biologically relevant, but were employed to provide highly stimulating environments that could induce metal decomplexation.

The [^64^Cu]Cu^2+^-labelled complexes were initially subjected to a transchelation experiment, employing DOTA as challenging agent (1000:1 DOTA-to-ligand molar ratio). As shown in Figure S8 and reported in Table 2, under these conditions, the [^64^Cu]Cu^2+^ complexes exhibited a noteworthy resistance to ligand-exchange, remaining >85% at the latest time point (24 h). A considerable drop in complex stability was observed only with DO3SAm.

Due to the high level of thiol-containing biomolecules in biological environments, any radiometal complex should be able to withstand transchelation to such molecules, thereby, effectively delivering the radiation to the molecular target. To probe this, the preformed [^64^Cu]Cu^2+^ complexes were incubated with a 1000-fold molar excess of cysteine (compared to the ligand) at physiological temperature (37 °C). As reported in Table 2, all the complexes showed relatively high stability as they remained >70% intact after 24 h.

### 2.4. Stability in Phosphate-Buffered Saline

Additional stability assays were performed to evaluate the integrity of the [^64^Cu]Cu^2+^ complexes obtained at low (i.e., 0.1 MBq/nmol) and high molar activity (i.e., 25 MBq/nmol) in phosphate-buffered saline (PBS). While all the ligands underwent experiments at low molar activity, the experiments at high molar activity were executed only for DO4S, DO2A2S, and DOTA (for comparison), since DO3S, DO3Sam, and TE4S showed to be unable to form complexes at 25 MBq/nmol.

**Figure 6:**
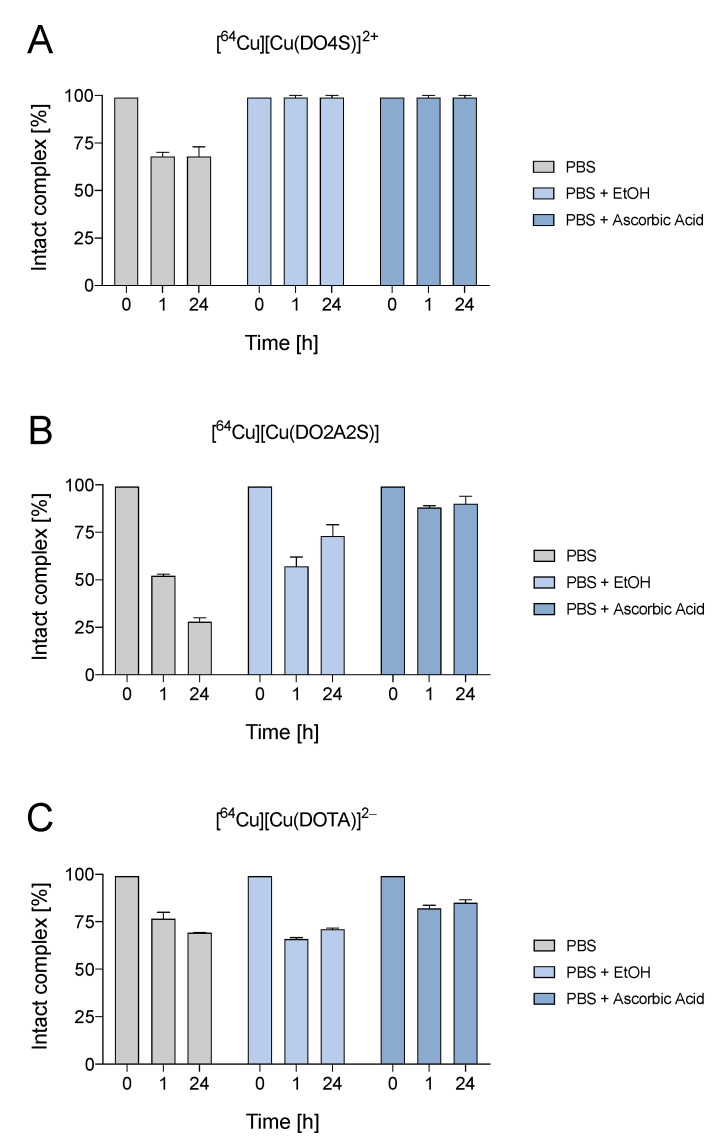
Stability of [^64^Cu]Cu^2+^-complexes prepared at 25 MBq/nmol in PBS with and without the additions of radioprotectants (ethanol 10% *v*/*v* and ascorbic acid 10% *v*/*v*). (**A**): [^64^Cu][Cu(DO4S)]^2+^; (**B**): [^64^Cu][Cu(DO2A2S)]; (**C**) [^64^Cu][Cu(DOTA)]^2−^.

The results obtained demonstrated that the [^64^Cu]Cu^2+^ complexes formed with all ligands at low molar activity have high stability in PBS, exhibiting >99% of intact complex over 24 h of incubation (Figure S9). Conversely, when high radioactive concentration was imposed, the complexes formed by the cyclen-based ligands tested were relatively stable (Figure 6), dropping significantly from 99% to ~70% for DO4S and ~25% for DO2A2S, respectively, after 24 h of incubation at ambient temperature. The observed degradation could be attributed to radiolysis, which triggers degradation over time.

Radiolysis quenchers, i.e., ethanol and ascorbic acid, were added to the PBS incubation solutions (Figure 6) to mitigate the radiation-induced degradation observed at high molar activities. The presence of radioprotectant led to a dramatic enhancement of the complex stability over time. For [^64^Cu][Cu(DO4S)]^2+^, no differences were found between ethanol and ascorbic acid that were both able to preserve the complex over 24 h, whilst for DO2A2S the highest improvement on the complex integrity (~90% after 24 h) was obtained using ascorbic acid. Interestingly, a roughly equivalent behavior was found for [^64^Cu][Cu(DOTA)]^2−^ (Figure 6) whereby it exhibits a stability comparable to [^64^Cu][Cu(DO4S)]^2+^ in the absence of radioprotectants and a marked improvement in the presence of ascorbic acid.

### 2.5. Human Serum Stability Assays

To gain insight into the stability and inertness of the [^64^Cu]Cu^2+^ complexes in a simulated biological environment over time, human serum stability assays were performed by incubating the preformed complex at 37 °C (DO3S, DO3SAm and TE4S were not considered for this study due to their poor radiolabelling ability at 25 MBq/nmol). The stability of the complex with DOTA was also assessed as a benchmark.

**Figure 7:**
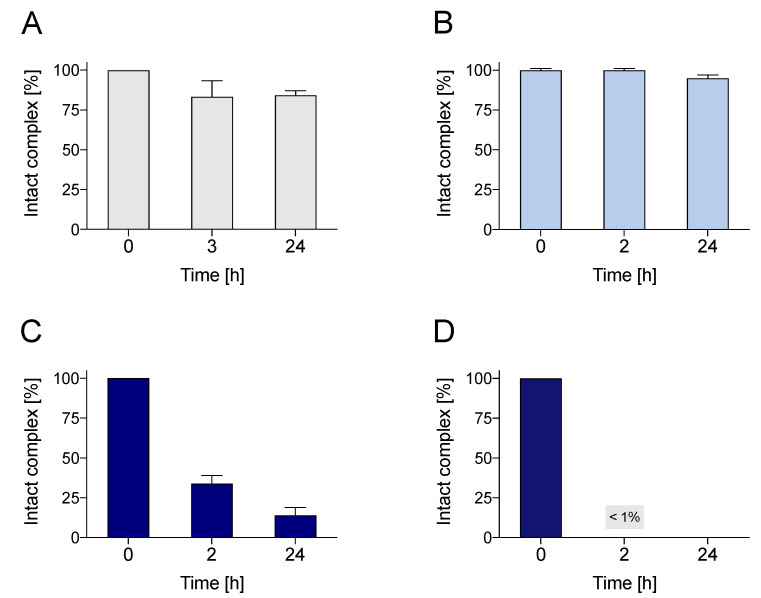
Human serum stability over time of (**A**) [^64^Cu][Cu(DOTA)]^2−^, (**B**) [^64^Cu][Cu(DO2A2S)], (**C**) [^64^Cu][Cu(DO4S)]^2+^ and (**D**) [^64^Cu][Cu(TRI4S)]^2+^.

As illustrated in Figure 7, [^64^Cu][Cu(DO2A2S)] was found to be highly stable in human serum with >99% of intact complex over 24 h. [^64^Cu][Cu(DOTA)]^2−^ showed slightly poorer overall integrity when compared with DO2A2S as it exhibited a stability of around 80%. Contrarily, just after 2 h, only ~30% and < 1% of the original radioactivity remained chelated to DO4S and TRI4S, respectively.

The poor stability in biologically relevant conditions experienced by [^64^Cu][Cu(DO4S)]^2+^ and [^64^Cu][Cu(TRI4S)]^2+^ is unexpected considering both the high thermodynamic stability of their cupric and cuprous complexes and the outcomes of the previous stability assays [1,33]. A possible explanation could be sought in the structure of these complexes in solution, as determined in our previous works: while with DO2A2S the metal center is fully encapsulated in a [4N]2O_ax_ octahedral structure, with DO4S and TRI4S the non-fully-saturated coordination sphere ([4N] + [4N]S_ax_ coordination) [1] could generate an open-labile site that favors the binding of competitive species, thus, provoking the observed demetallation.

### 2.6. Small-Animal Imaging, Biodistribution and In Vivo Stability

**Figure 8:**
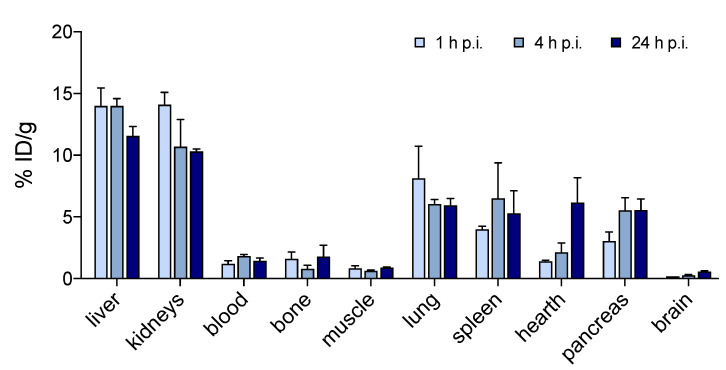
[^64^Cu][Cu(DO2A2S)] biodistribution in nude BALB/C mice at 1, 4, and 24 h post injection by tail vein. Data are presented as mean ± SEM (*n* = 3/group).

**Figure 9:**
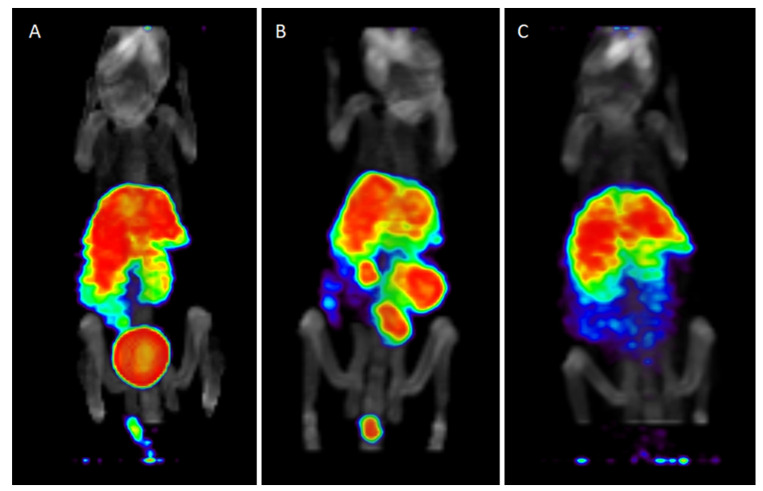
Representative PET/CT images of [^64^Cu][Cu(DO2A2S)] injected in nude BALB/C mice at (**A**) 1 h, (**B**) 4 h and (**C**) 24 h post-injection by tail vein (%ID/voxels).

Among all the chelators investigated, the data collected to date indicate DO2A2S to exhibit the most promising results in terms of kinetics and radiolabelling conditions. This is sustained by the extremely high stability of [^64^Cu][Cu(DO2A2S)] in physiological media (Figure 7). These findings prompted us to investigate the [^64^Cu][Cu(DO2A2S)] biodistribution in BALB/c nude mice and its stability in biological fluid (urine and blood) at 1, 4, and 24 h post-injection. Organ uptake and clearance pathways were also qualitatively evaluated by means of PET/CT. The results of these examinations are shown in Figure 8 and Figure 9.

[^64^Cu][Cu(DO2A2S)] displayed a metabolic trend similar to other [^64^Cu]-labelled cyclen-based derivatives [37,38,39]. At 1 h post-injection, most of the radioactivity was associated with the liver and kidneys (14.0 and 14.1% ID/g, respectively), but relatively high accumulation was found in the lungs, spleen and pancreas as well (8, 4, and 3% ID/g, respectively). [^64^Cu][Cu(DO2A2S)] exhibited fast blood clearance with blood levels < 2% ID/g while the retention in bone and muscle was almost negligible (<2 and <1% ID/g, respectively).

**Figure 10:**
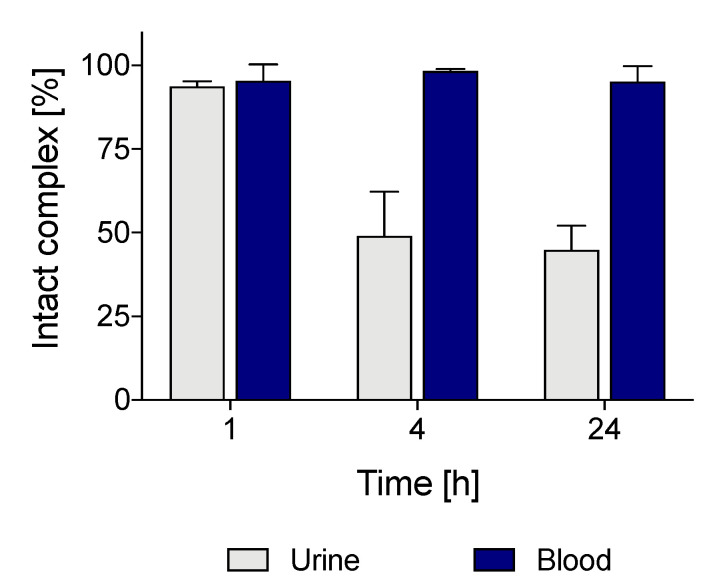
[^64^Cu][Cu(DO2A2S)] stability in urine and blood samples collected from mice at 1, 4, and 24 h post injection. Data are presented as mean ± SEM (*n* = 3/group).

The rapid blood clearance suggested that [^64^Cu][Cu(DO2A2S)] did not appreciably dissociate in the blood. This finding was confirmed by stability studies performed on blood samples withdrawn at each time point, which revealed the formation of less than 5% free copper-64 within 24 h from the injection (Figure 10).

Liver, kidneys, and lungs cleared slowly with 82, 73, and 75% of the dose found at 1 h remaining in the organs at 24 h post-injection, respectively. A progressive accumulation over time was observed in the spleen and pancreas (up to 5.3 and 5.5% ID/g).

A prolonged retention of radioactivity in liver and kidneys is often associated with a reduced complex stability due to copper biological reduction followed by transchelation phenomena [22,38,40]. These processes are likely mediated by superoxide dismutase (SOD1), a dimeric enzyme mainly expressed in liver, kidneys, and erythrocytes cells [19,41]. Moreover, the liver is commonly known as the major captor, distributor, and excretory of copper ions [42]. However, the accumulation of radioactivity in this organ is not unequivocally synonymous with complex cleavage. For instance, it has been reported that in mice injected with a [^64^Cu]Cu-labelled cyclam derivative, the high liver uptake was due to intrinsic properties of the compound and not to copper release or transchelation [5]. The different overall charges, namely, lipophilicity and chemical moieties forming the structure of the complex, play a fundamental role in the biodistribution [40,43,44,45]. It should be noted that the [^64^Cu]Cu^2+^ complex formed by DO2A2S is neutral at physiological pH [1] while, for example, the complexes formed by DOTA and other carboxylate-based ligands are negatively charged (e.g., −2 for DOTA) [1].

Thus, [^64^Cu][Cu(DO2A2S)] might be markedly more lipophilic than [^64^Cu][Cu(DOTA)]^2−^, explaining the highest hepatic metabolization and elimination [46]. Moreover, the presence of sulfur-containing pendant arms in DO2A2S likely plays a pivotal role in the metabolic behavior of its [^64^Cu]Cu^2+^ complexes and could be a reason for its high liver accumulation and retention. Sulfur-containing compounds deal with quite complicated metabolic pathways in mammals but trans-sulfuration is usually engaged by hepatic enzymes [46]. In spite of these considerations, the studies performed so far are not sufficient for elucidating whether transchelation actually occurred or the high accumulation in the liver is simply due to metabolic derivatives of intact complex.

On the other hand, the high level of intact complex in blood at 24 h post injection and, conversely, the presence of almost 50% of free-[^64^Cu]Cu^2+/+^ in urine samples already at 4 h post-injection, lead to postulate that the cleaved fraction of [^64^Cu][Cu(DO2A2S)] is mainly excreted by the kidneys.

## 3. Materials and Methods

### 3.1. Materials

All chemicals purchased from commercial suppliers were of analytical grade or higher and were used as received without further purification. Ultrapure water (18.2 MΩ/cm, Millipore Milli-Q/plus) was used. Furthermore, 1,4,7,10-Tetrakis[2-(methylsulfanyl)ethyl]-1,4,7,10-tetraazacyclododecane (DO4S), 1,4,7-tris[2-(methylsulfanyl)ethyl]-1,4,7,10-tetraazacyclododecane (DO3S), 1,4,7-tris[2-(methylsulfanyl)ethyl]-10-acetamido-1,4,7,10-tetraazacyclododecane (DO3SAm), 1,7-bis[2-(methylsulfanyl)ethyl]-4,10,diacetic acid-1,4,7,10-tetraazacyclododecane (DO2A2S), 1,4,7,10-tetrakis[2-(methylsulfanyl)ethyl]-1,4,7,10-tetrazacyclotridecane (TRI4S) and 1,4,8,11-tetrakis[2-(methylsulfanyl)ethyl]-1,4,8,11-tetrazacyclotetradecane (TE4S) were synthesized according to previously reported procedures [32,33]. Moreover, 1,4,7,10-Tetraazacyclododecane-1,4,7,10-tetraacetic acid (DOTA) was obtained from Chematech, Dijon, France. NODAGA-RGD (NODAGA-RGD trifluoroacetate) was obtained from ABX GmbH, advanced biochemical compounds, Radeberg, Germany.

### 3.2. Copper-64 Production

Copper-64 chloride ([^64^Cu]CuCl_2_) in 0.5 M HCl was purchased from Advanced Center Oncology Macerata—ACOM (Macerata, Italy). Alternatively, it was produced at Paul Scherrer Institute (PSI, Villigen, Switzerland) via the ^64^Ni(p,n)^64^Cu reaction. Enriched nickel-64 coated gold targets were irradiated with protons degraded to 11 MeV at PSI’s Inject 2 72 MeV research cyclotron and purified with an especially-designed separation panel located into a dedicated hot cell following a previously published chemical separation procedure [34]. After the separation, the molar activity of the ^64^Cu-solution in 0.05 M HCl was around 40 GBq/nmol.

### 3.3. Copper-64 Radiolabelling

Ligand stock solutions were prepared in ultrapure water at 10^−3^ M and were kept in the freezer when not used. Serial dilutions were performed to obtain solutions with ligand concentrations ranging from 1.0⋅10^−4^ M to 1.0⋅10^−7^ M in ultrapure water. Diluted solutions were freshly prepared from stock solutions before each experiment.

Radiolabelling were performed by adding [^64^Cu]CuCl_2_ diluted in 0.05 M HCl (~2.5 MBq, 2 μL) to an aliquot of ligand solution (20 μL) mixed with 0.05 M HCl + 0.5 M sodium acetate 5:1 *v*/*v* ratio (98 μL and 20 μL, respectively) [47]. The final pH was equal to 4.5. In the experiments performed at pH 7, 1.0 M sodium phosphate buffer (100 μL) was used instead of sodium acetate buffer. The reaction mixtures were allowed to react for 10 min at RT or heated to 90 °C. Different ligand concentrations were screened, which correspond to the following molar activities: 2 × 10^−3^ M (0.01 MBq/nmol), 2·10^−4^ M (0.1 MBq/nmol), 2 × 10^−5^ M (1 MBq/nmol), 8 × 10^−6^ M (2.5 MBq/nmol), 4·× 10^−6^ M (5 MBq/nmol), 2·× 10^−6^ M (10 MBq/nmol), 8·× 10^−7^ M (25 MBq/nmol), 4·× 10^−7^ M (50 MBq/nmol), 2·× 10^−7^ M (100 MBq/nmol), 8·× 10^−8^ M (250 MBq/nmol) and 4·× 10^−8^ M (500 MBq/nmol). All measurements were performed in triplicates.

The reaction progress was monitored by radio-ultra-high-performance liquid chromatography (UHPLC) or radio-thin layer chromatography (TLC). UHPLC was performed using an Acquity system (Waters, Italy) equipped with a reversed-phase C18 column (1.7 μm, 2.1 mm × 150 mm), an Acquity TUV detector (Waters, Milan, Italy) and a Herm LB 500 radiochemical detector (Berthold Technologies, Milan, Italy). 0.1% Trifluoroacetic acid in water (A) and acetonitrile (B) composed the mobile phase. The elution gradient included three stages. A was kept constant to 5% for 2 min; then a 5-min gradient, from 5 to 25% of A, was used; finally, the initial condition was restored in 2 min. A flow rate of 0.35 mL/min was employed. The [^64^Cu]Cu^2+^ complexes were retained at characteristic retention time (t_R_) equal to: ~6.9 min for [^64^Cu][Cu(DO4S)]^2+^, ~5.8 min for [^64^Cu][Cu(DO3S)]^2+^, ~6.8 min for [^64^Cu][Cu(DO3SAm)]^2+^, ~4.0 min for [^64^Cu][Cu(DO2A2S)], ~6.8 min for [^64^Cu][Cu(TRI4S)]^2+^ and ~6.7 min for [^64^Cu][Cu(TE4S)]^2+^. Free [^64^Cu]Cu^2+^ eluted at t_R_~1 min.

TLC silica gel 60 F254 plates, developed using a mixture of 10% ammonium acetate and methanol (1:1 *v*/*v* ratio, pH 5.5), were used as stationary phase with DO2A2S, DOTA and NODAGA-RDG [47]. Under these conditions, the free [^64^Cu]Cu^2+^ remains at the baseline (retention factor, R_f_ = 0) while the [^64^Cu]Cu^2+^ complexes migrate with the liquid phase (R_f_~0.6 for [^64^Cu]Cu-NODAGA-RDG and R_f_~0.4 for [^64^Cu][Cu(DO2A2S)]). Silica gel 60 RP-18 F254S plates were employed as stationary phase and sodium citrate (1 M, pH 4) as eluent with DO4S, TRI4S and TE4S. Under these conditions, the unbound [^64^Cu]Cu^2+^ migrates with the solvent front (R_f_ = 1) while the [^64^Cu]Cu^2+^ complexes remain at the baseline (R_f_ = 0).

TLC plates were analyzed using a Cyclone Plus Storage Phosphor System (Perkin Elmer) after exposure to a super-resolution phosphor screen (Type MS, Perkin Elmer, Waltham, MA, USA). All the data were processed with OptiQuant software (version 5.0, Perkin Elmer Inc., Waltham, MA, USA).

### 3.4. Competition Assays

Competitions studies with DOTA were performed by labelling [^64^Cu]Cu^2+^ with chelators (0.5 MBq/nmol, pH 4.5 or pH 7, RT, 10 min) in the presence of a 1:1 DOTA-to-ligand ratio. Metal competition studies were performed by adding the ligand (0.5 MBq/nmol, pH 4.5, RT, 10 min) in a mixture containing [^64^Cu]Cu^2+^ and a 2:1 metal-to-ligand excess of Ni^2+^ or Zn^2+^. Competition assays among the chelators were carried out by mixing a solution containing equimolar amounts of chelators with [^64^Cu]Cu^2+^ (0.5 MBq/nmol, pH 4.5, RT). The reaction mixtures were analyzed by UHPLC, using the method described above.

### 3.5. Stability Assays with DOTA and Cysteine

The stability of the preformed [^64^Cu]Cu^2+^ complexes was assessed by adding a 1000:1 DOTA/cysteine-to-ligand molar ratio excess. The incubation temperature was set at 37 °C when cysteine was used to simulate the biological environment, whilst it was kept at RT when DOTA was employed as a challenging agent.

The complex stability was evaluated over time by withdrawing an aliquot from the reaction mixtures. The latter was analyzed by UHPLC using the method described above.

### 3.6. Stability in Phosphate-Buffered Saline

The stability of the preformed [^64^Cu]Cu^2+^ complexes in phosphate-buffered saline (PBS) was explored by diluting the labelling solution 1:1 *v*/*v* in PBS (pH 7.4). Both low and high molar activities (0.1 MBq/nmol and 25 MBq/nmol, respectively) were employed to evaluate the effect of radiolysis. Radioprotectants (ethanol 10% *v*/*v* or ascorbic acid 10% *v*/*v*) were added to PBS to quench the radiolysis phenomena [47]. The [^64^Cu]Cu^2+^ complex stabilities were investigated at different time points after preparation using the TLC or UHPLC protocols reported previously.

### 3.7. Human Serum Stability

Human serum stability assays were performed as follow: the preformed [^64^Cu]Cu^2+^ complexes (prepared at 25 MBq/nmol) were diluted 1:5 *v*/*v* in human serum. The samples were incubated at 37 °C to simulate the biological environment. At the defined time point, 50 μL of the plasma-containing mixture were added to 200 μL of ice-cold methanol (0.16 MBq/μL final concentration) and centrifuged for 3 min at 8000 rpm. Afterwards, 2 μL of supernatant were spotted on TLC plates developed using the previous protocols. Dissociation of [^64^Cu]Cu^2+^ from the complexes was also monitored at varying time points using the same procedure with the exception that no proteins were precipitated. In this case, 2 μL of human plasma containing the labelled complex was directly spotted on the TLC plate. No differences in the percentage of intact complexes were observed with or without protein precipitation. Both procedures were validated by incubating free [^64^Cu]Cu^2+^ with human plasma.

### 3.8. Small Animal Imaging, Biodistribution and Stability

All procedures involving animals were performed in accordance with ethical protocols approved by the Italian Ministry of Health (n. 44/2021-PR). Six-week-old female BALB/c nude mice (*n* = 9) were purchased from Charles River/Envigo and divided into three groups. Mice (*n* = 3/group) were administered with 7–14 MBq (depending on acquisition time after injection) of [^64^Cu][Cu(DO2A2S)] solution by caudal intravenous injection. The exact amount of injected radioactivity was determined using a Capintec CRC-15PET dose calibrator. Micro PET/CT data were collected at 1, 4, and 24 h post injection using a Bruker Albira Si. Mice were anaesthetized with 1–2% isoflurane in a flow of oxygen (2 L/min 100% O_2_) and images were acquired over 20 min (10 min for PET and CT images acquisition, respectively). PET data were reconstructed using MLEM GPU (Graphics Processing Unit), while Filter Back Projection Algorithm was used for reconstructing CT images. Mice were euthanized by cervical dislocation immediately following the PET/CT scan and organs and tissues of interest were collected and weighed. Radioactivity associated with organs was measured using a γ-spectrometer based on a 1″×1″ inch cylindrical LBC (Lanthanum BromoChloride), LaBr_2.85_Cl_0.15_:Ce, inorganic scintillator manufactured by SCIONIX coupled to a HAMAMATSU R11102 Photomultiplier tube (PMT) which was previously characterized and validated against a Capintec CRC-15PET dose calibrator [48]. The results were corrected for decay to the time of injection. Radioactivity values were expressed as percentage of injected dose (mean ± SEM) per gram of tissue or organ (% ID/g). Concurrently, blood and urine aliquots (1 μL) were sampled for stability tests, which were carried out by radio-TLC scans (Cyclone, Perkin Elmer) by using silica gel 60 RP-18 F254S plates and 0.1 M sodium citrate as eluent.

## 4. Conclusions

The stabilization of redox-active copper ions in vivo represents a challenge for developing stable and safe (radio)pharmaceuticals as the biologically activated Cu^2+^-Cu^+^ reduction can trigger the metal release from the chelate. To hamper this undesired phenomenon, we have developed a series of S-rich polyazamacrocycles with different pendant arms and backbone sizes. The thermodynamic data indicated a remarkable stability of both their cupric and cuprous complexes, and the structural analysis demonstrated that a switch can occur among donor atoms upon copper reduction: for example, for DO2A2S, Cu^2+^ and Cu^+^ coordinating donors are [4N,2O] and [4N,1S], respectively [1]. Hereby, their radiolabelling ability toward [^64^Cu]Cu^2+^, and the in vitro/in vivo stability of the corresponding [^64^Cu]Cu^2+^ complexes were explored.

Radiolabelling experiments demonstrated a large gap between the ability of each ligand to complex [^64^Cu]Cu^2+^. Among the cyclen-based ligands, DO2A2S possessed the highest affinity for copper, as it labelled at high molar activities (50 MBq/nmol) under mild reaction conditions, which fulfils the need for pH- and thermally sensitive tumor-targeting vectors (RT, 10 min, pH 7) such as, for instance, antibodies. [^64^Cu][Cu(DO2A2S)] exhibited excellent human serum stability over a 24 h period. Contrarily, when the carboxylic chains were replaced with sulfur ones, DO4S possessed inferior labelling behavior, as well as poorer human serum integrity. The same trend was observed with DO3S and DO3SAm. Among the non-cyclen based ligands, TRI4S showed a comparable labelling behavior with respect to DO4S, but the stability of the resulting complex was poorer. A further increase in the ring size to 14-member macrocyclic derivative (TE4S) resulted in an evident drop in the labelling ability, making any further evaluation of the stability of its [^64^Cu]Cu^2+^ complex nonviable.

These results highlight the importance of considering both the proper backbone and the presence of the carboxylic donors, as these features have demonstrated a noteworthy impact on the chelator performance and the [^64^Cu]Cu^2+^ complex stability in biological media.

The in vivo investigation conducted hitherto on the most promising [^64^Cu]Cu^2+^ complex showed that [^64^Cu][Cu(DO2A2S)] displayed a metabolic trend similar to other [^64^Cu]-labelled cyclen-based derivatives reported in the literature with high accumulation and persistent retention in the kidneys and liver [37,38,39].

The promising properties demonstrated by DO2A2S warrant the further development of a bifunctional derivative bound to tumor-targeting vectors. As reported above, we previously found that this ligand coordinates Cu^2+^ and Cu^+^ through a [4N,2O] and [4N,1S] donor moiety, respectively. To retain its coordination properties, the bifunctional derivative should preserve the two acetate donors to allow Cu^2+^ complexation, and at least one sulfur donor in an attempt to obstruct the possible release of Cu^+^ upon in vivo reduction.

## Figures and Tables

**Table 1:** Labelling efficiency (%) in the presence of a molar excess (2-fold with respect to the chelator) of competitive metal ions.

	Zn^2+^	Ni^2+^
[^64^Cu][Cu(DO4S)]^2+^	100 ± 1	100 ± 2
[^64^Cu][Cu(DO3S)]^2+^	95 ± 2	91 ± 2
[^64^Cu][Cu(DO3SAm)]^2+^	90 ± 2	99 ± 2
[^64^Cu][Cu(DO2A2S)]	64 ± 5	73 ± 3
[^64^Cu][Cu(TRI4S)]^2+^	100 ± 1	100 ± 1

**Table 2:** Time-dependent stability of [^64^Cu]Cu^2+^ complexes in the presence of DOTA (1000: 1 DOTA-to-ligand molar ratio) and cysteine (1000:1 Cys-to-ligand molar ratio). Values are represented as % of intact complex. Uncertainties are in the order of ± 2% and are omitted for sake of clarity.

Complex	DOTA			Cysteine		
	0 h	7 h	24 h	0 h	3 h	24 h
[^64^Cu][Cu(DO4S)]^2+^	100	92	88	100	100	83
[^64^Cu][Cu(DO3S)]^2+^	100	100	100	100	100	100
[^64^Cu][Cu(DO3SAm)]^2+^	100	80	38	100	83	72
[^64^Cu][Cu(DO2A2S)]	91	90	87	100	85	74
[^64^Cu][Cu(TRI4S)]^2+^	−	−	−	−	−	−

## Data Availability

Data are available by the corresponding author upon request.

## Supplementary Materials

The following supporting information can be downloaded at: https://www.mdpi.com/article/10.3390/molecules27134158/s1, Figure S1: Paradigmatic radio-chromatograms of quantitative [64Cu]Cu2+-labelled cyclen-based ligands (1 MBq/nmol); Figure S2: Paradigmatic radio-chromatograms of [64Cu]Cu2+-labelled non-cyclen based ligands (1 MBq/nmol); Figure S3: Time-dependent RCIs for [64Cu]Cu2+ radiolabelling at pH 4.5 and RT with (A) DO4S (10 MBq/nmol) and (B) DO3S (5 MBq/nmol); Figure S4: Radio-chromatograms of the DOTA competition assays (1:1 DOTA-to-ligand molar ratio) with (A) DO4S, (B) DO3S, (C) DO3SAm and (D) DO2A2S at pH 4.5 and RT; Figure S5: Radio-chromatograms of the DOTA competition assays (1:1 DOTA-to-ligand molar ratio) with (A) DO4S, (B) DO3S, (C) DO3SAm and (D) DO2A2S at pH 7 and RT; Figure S6: Radio-chromatograms related to the competition assay among the cyclen-based S-rich chelators (at equimolar amounts) at pH 4.5 and RT; Figure S7: Percentage of the different [64Cu]Cu2+ complexes obtained in the challenge assay at different time points; Figure S8: Radio-chromatograms of the DOTA stability assay (1000:1 DOTA-to-ligand molar ratio). (A) [64Cu][Cu(DO4S)]2+, (B) [64Cu][Cu(DO3S)]2+, (C) [64Cu][Cu(DO3SAm)]2+ and (D) [64Cu][Cu(DO2A2S)]; Figure S9: Radio-chromatograms of the PBS stability assays at low molar activity. (A) [64Cu][Cu(DO4S)]2+, (B) [64Cu][Cu(DO3S)]2+, (C) [64Cu][Cu(DO3SAm)]2+ and (D) [64Cu][Cu(DO2A2S)]; Table S1. [64Cu]Cu2+ incorporation in the cyclen-based ligands and NODAGA-RGD at several molar activities (pH 4.5); Table S2. [64Cu]Cu2+ incorporation in the cyclen-based ligands and NODAGA-RGD at several molar activities (pH 7.0); Table S3. [^64^Cu]Cu^2+^ incorporation in the non-cyclen-based ligands and NODAGA-RGD at several molar activities (pH 7.0).

## Sample Availability

Samples of DO4S, DO3S, DO3SAm, DO2A2S, TRI4S and TE4S are available from the authors.

## Abbreviations

1,4,7,10-Tetraazabicyclo[5.5.2]tetradecane-4,10-diacetic acid (CB-DO2A), 1,4,8,11-tetraazabicyclo[6.6.2]hexadecane-4,11-diacetic acid (CB-TE2A), 1,7-bis[2-(methylsulfanyl)ethyl]-4,10,diacetic acid-1,4,7,10-tetraazacyclododecane (DO2A2S), 1,4,7-tris[2-(methylsulfanyl)ethyl]-1,4,7,10-tetraazacyclododecane (DO3S), 1,4,7-tris[2-(methylsulfanyl)ethyl]-10-acetamido-1,4,7,10-tetraazacyclododecane (DO3SAm), 1,4,7,10-tetrakis[2-(methylsulfanyl)ethyl]-1,4,7,10-tetraazacyclododecane (DO4S), 1,4,7,10-tetraazacyclododecane-1,4,7,10-tetraacetic acid (DOTA), 1,4,7-triazacyclononane,1-glutaric acid-4,7-acetic acid (NODAGA), 1,4,7-triazacyclononane-1,4,7-triacetic acid (NOTA), Phosphate-Buffered Saline (PBS), Positron Emission Tomography (PET), Radiochemical Incorporation (RCI), Superoxide Dismutase (SOD1), 1,4,8,11-tetrakis[2-(methylsulfanyl)ethyl]-1,4,8,11-tetrazacyclotetradecane (TE4S), 1,4,8,11-tetraazacyclotetradecane-1,4,8,11-tetraacetic acid (TETA), 1,4,7,10-tetrakis[2-(methylsulfanyl)ethyl]-1,4,7,10-tetrazacyclotridecane (TRI4S), Thin-Layer Chromatography (TLC), Ultra-High-Performance Liquid Chromatography (UHPLC).
