# Supplementary material for: Chelation of Theranostic Copper Radioisotopes with S-Rich Macrocycles: From Radiolabelling of Copper-64 to In Vivo Investigation
Source: Molecules. 2022 Jun 28;27(13):4158. doi: 10.3390/molecules27134158 (PMC9268100; doi:10.3390/molecules27134158)
Supplement: Supplementary file 1 [file molecules-27-04158-s001.zip › molecules-1792129-supplementary.pdf]

Supporting Information

Chelation of Theranostic Copper Radioisotopes with S-Rich Macrocycles: From Radiolabelling of Copper-64 to *In Vivo* Investigation

Marianna Tosato[1], Marco Verona[2], Chiara Favaretto[3], Marco Pometti[4], Giordano Zanoni[1], Fabrizio Scopelliti[4], Francesco Paolo Cammarata[5], Luca Morselli[6], Zeynep Talip[3], Nicholas P. van der Meulen[3,7], Valerio Di Marco[1], Mattia Asti[8,*]

[1] Department of Chemical Sciences, University of Padova, via Marzolo 1, 35131 Padova, Italy; marianna.tosato@unipd.it (M.T.); giordano.zanoni@unipd.it (G.Z.); valerio.dimarco@unipd.it (V.D.M.)

[2] Department of Pharmaceutical Sciences, University of Padova, via Marzolo 8, 35131 Padova, Italy; marco.verona@phd.unipd.it (M.V.)

[3] Center for Radiopharmaceutical Sciences, Paul Scherrer Institute, 5232 Villigen-PSI, Switzerland; chiara.favaretto@psi.ch (C.F); zeynep.talip@psi.ch (Z.T.); nick.vandermeulen@psi.ch (N.V.D.M.)

[4] Nuclear Medicine Department, Cannizzaro Hospital, via Messina 829, 95126 Catania, Italy; marco.pometti@gmail.com (M.P.); fabrizioscopelliti@gmail.com (F.S.)

[5] Institute of Molecular Bioimaging and Physiology, National Research Council (IBFM-CNR), 90015 Cefalù, Italy; francesco.cammarata@ibfm.cnr.it (F.P.C.)

[6] Italian Institute of Nuclear Physics, Legnaro National Laboratories, viale dell'Università 2, 35020 Legnaro, Padova, Italy; luca.morselli@lnl.infn.it (L.M.)

[7] Laboratory of Radiochemistry, Paul Scherrer Institute, 5232 Villigen-PSI, Switzerland

[8] Radiopharmaceutical Chemistry Section, Nuclear Medicine Unit, AUSL-IRCCS di Reggio Emilia, via Amendola 2, 42122 Reggio Emilia, Italy; mattia.asti@auls.re.it (M.A.)

* **Correspondence**: mattia.asti@auls.re.it

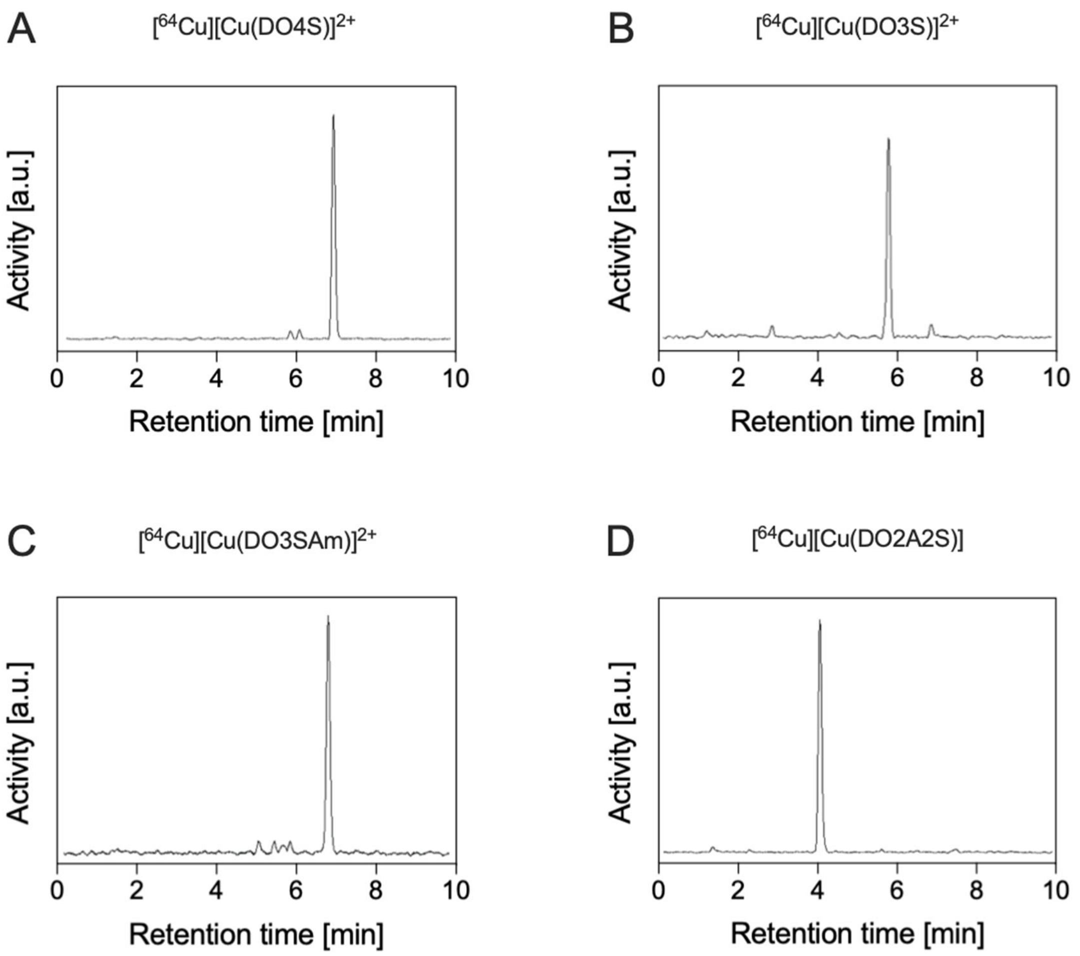

Figure S1. Paradigmatic radio-chromatograms of quantitative [^{64}Cu]Cu^{2+}-labelled cyclen-based ligands (1 MBq/nmol).

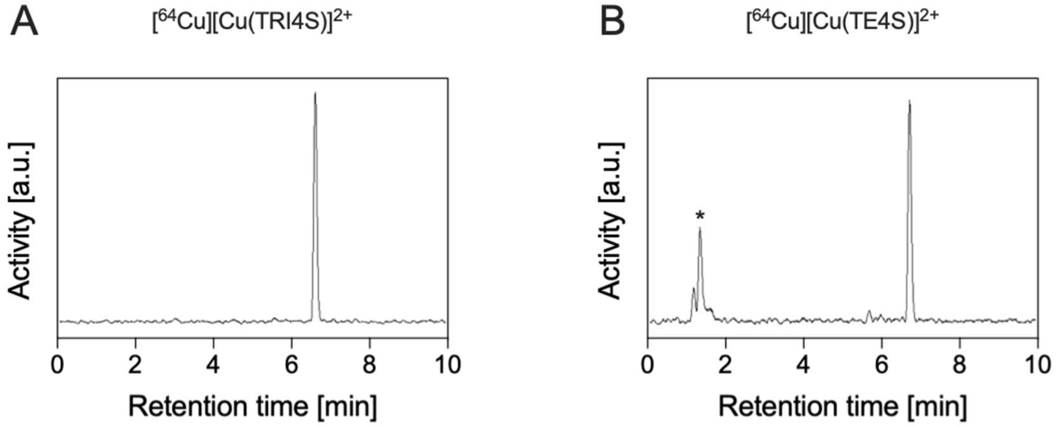

Figure S2. Paradigmatic radio-chromatograms of [^{64}Cu]Cu^{2+}-labelled non-cyclen based ligands (1 MBq/nmol). The peak marked with an asterisk is related to unbound [^{64}Cu]Cu^{2+}.

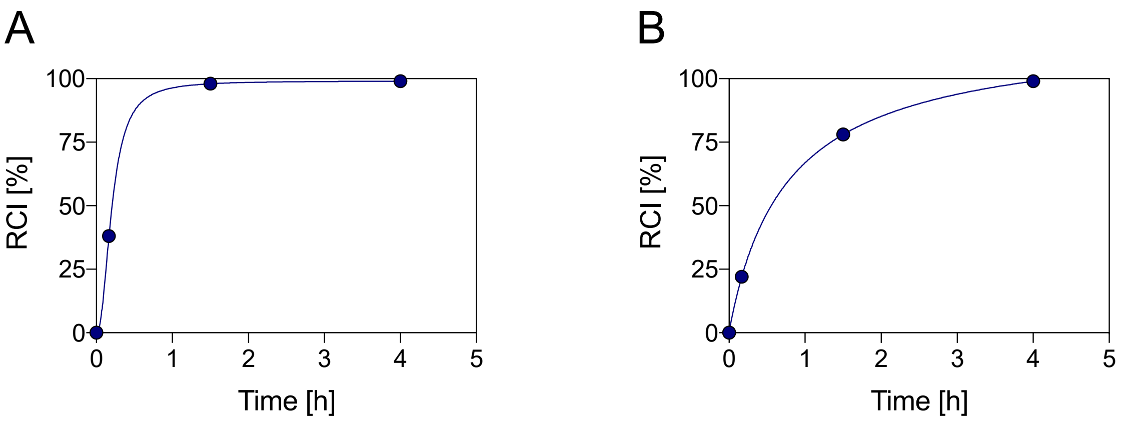

Figure S3. Time-dependent RCIs for $[^{64}Cu]Cu^{2+}$ radiolabelling at pH 4.5 and RT with (A) DO4S (10 MBq/nmol) and (B) DO3S (5 MBq/nmol).

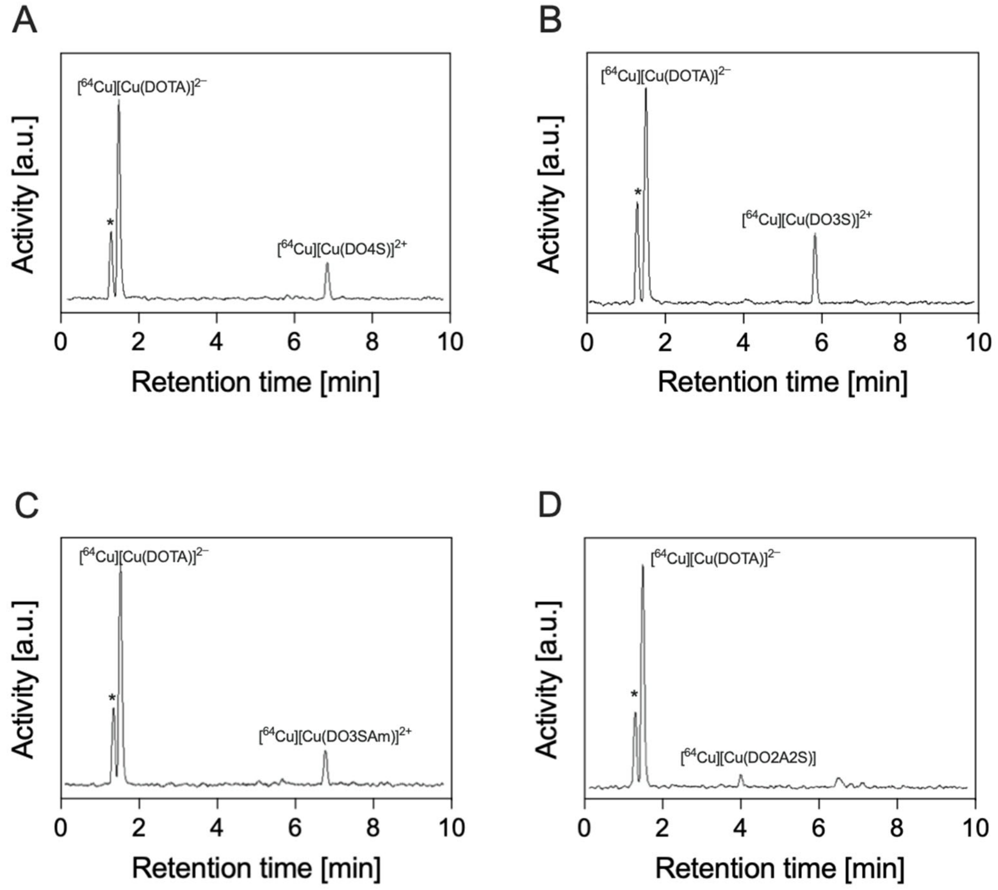

Figure S4. Radio-chromatograms of the DOTA competition assays (1:1 DOTA-to-ligand molar ratio) with (A) DO4S, (B) DO3S, (C) DO3SAm and (D) DO2A2S at pH 4.5 and RT. The peaks marked with an asterisk show free $[^{64}Cu]Cu^{2+}$.

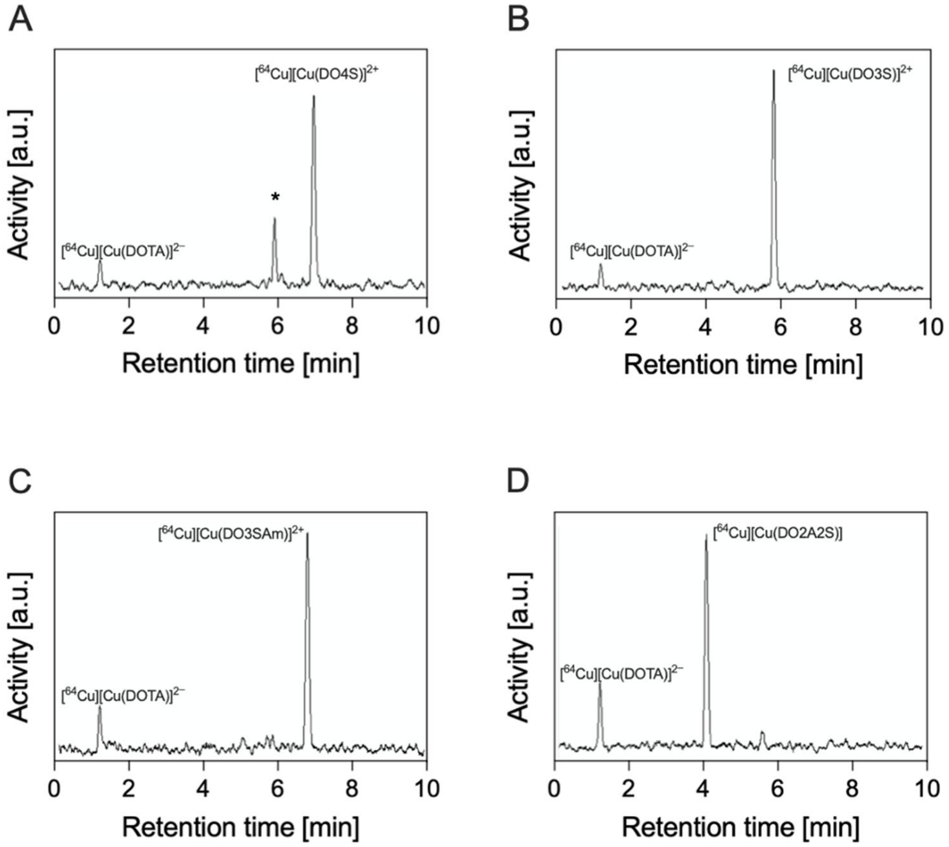

Figure S5. Radio-chromatograms of the DOTA competition assays (1:1 DOTA-to-ligand molar ratio) with (A) DO4S, (B) DO3S, (C) DO3SAm and (D) DO2A2S at pH 7 and RT. The peak marked with an asterisk shows an impurity of DO3S.

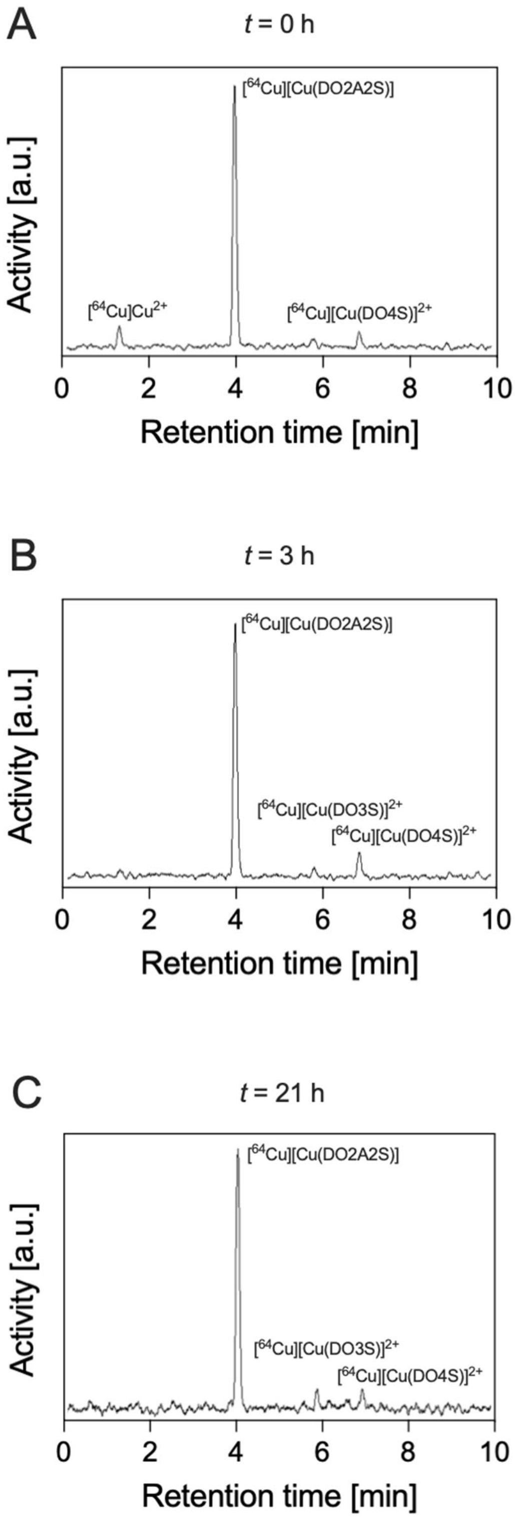

Figure S6. Radio-chromatograms related to the competition assay among the cyclen-based S-rich chelators (at equimolar amounts) at pH 4.5 and RT.

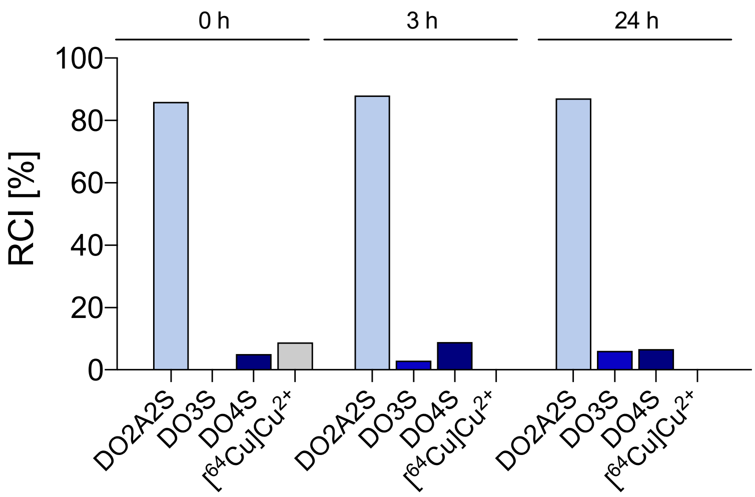

Figure S7. Percentage of the different [^{64}Cu]Cu^{2+} complexes obtained in the challenge assay at different time points. Data are referred to **Figure S6**.

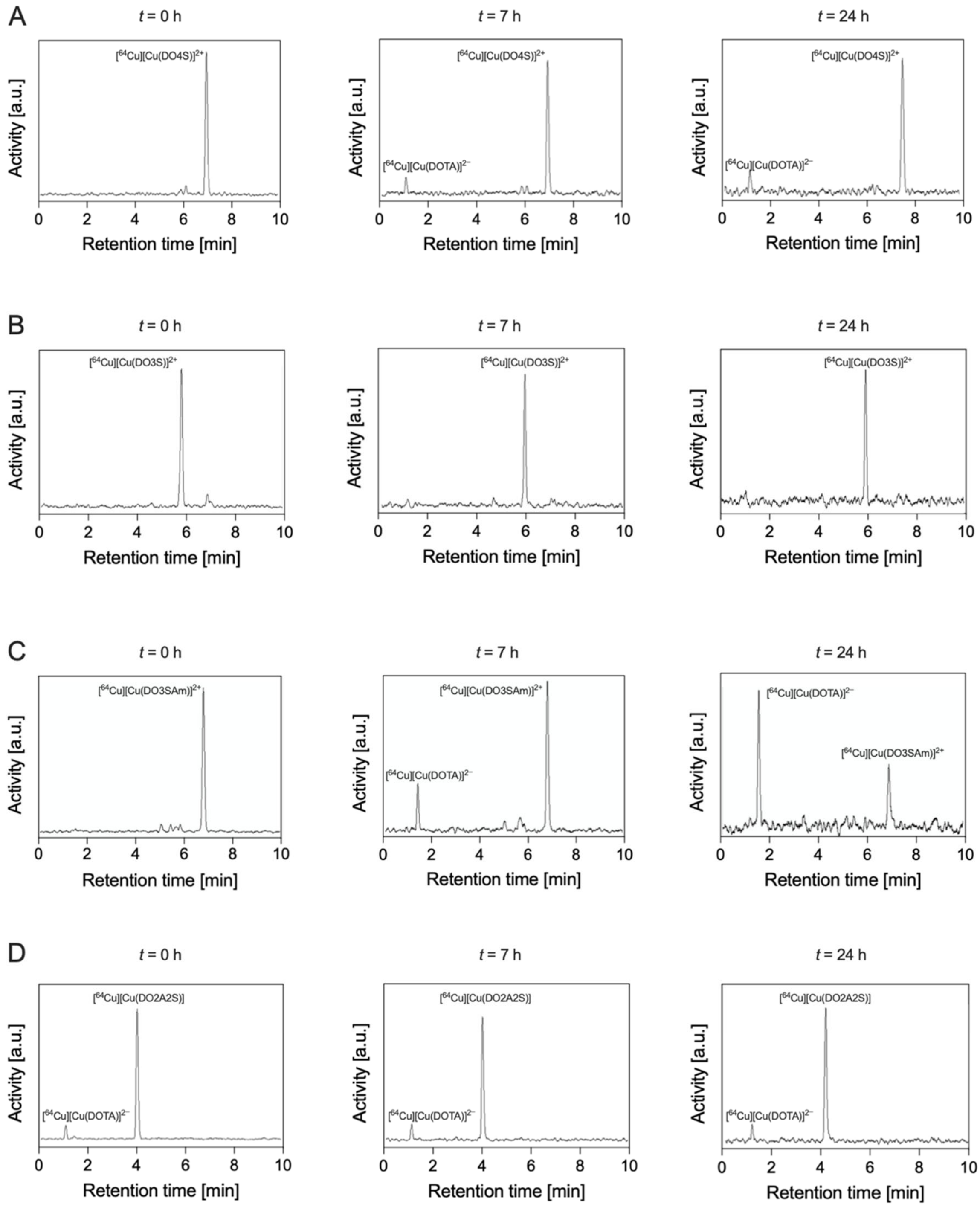

Figure S8. Radio-chromatograms of the DOTA stability assay (1000:1 DOTA-to-ligand molar ratio). (A) [^{64}Cu][Cu(DO4S)]$^{2+}$, (B) [^{64}Cu][Cu(DO3S)]$^{2+}$, (C) [^{64}Cu][Cu(DO3SAm)]$^{2+}$ and (D) [^{64}Cu][Cu(DO2A2S)].

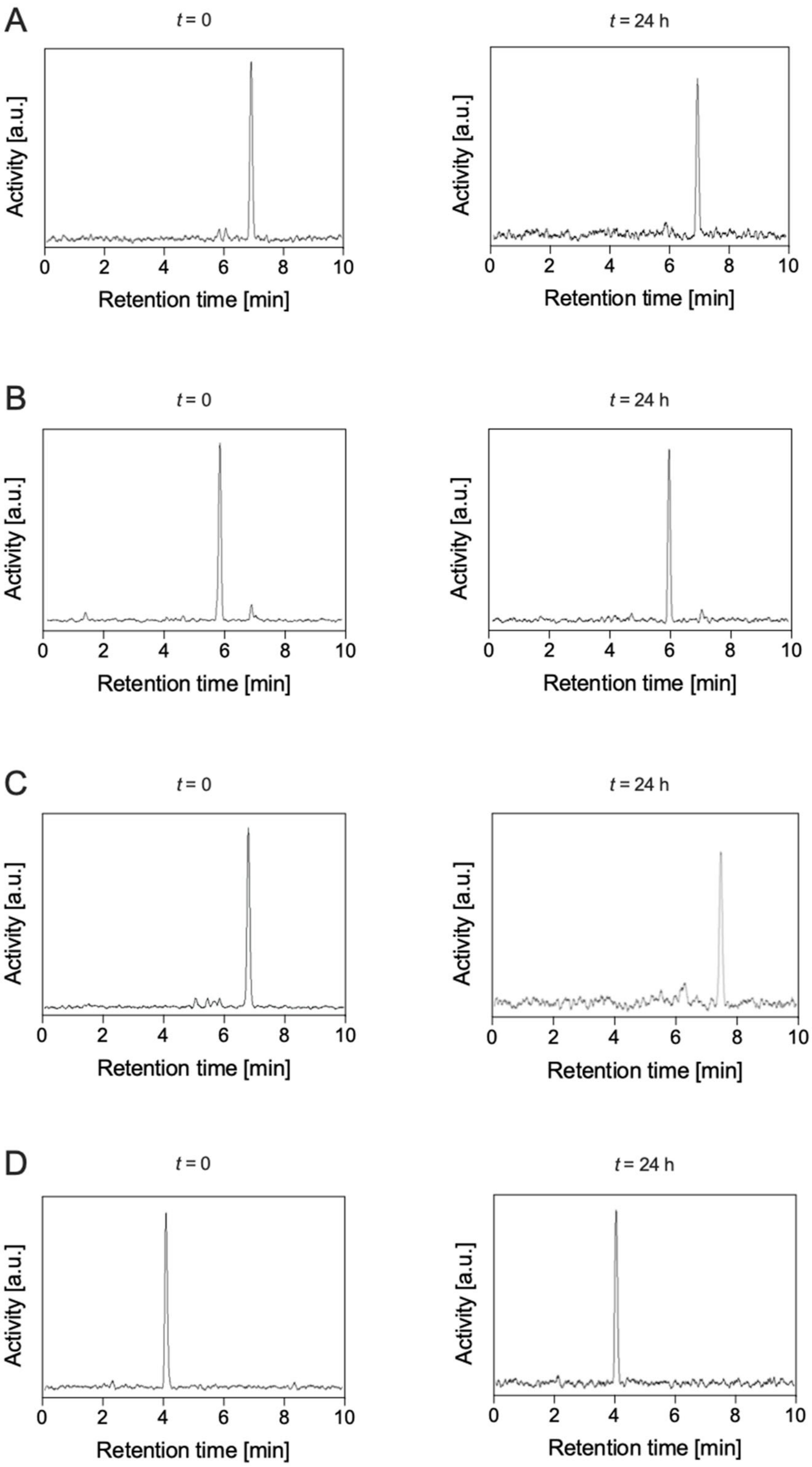

Figure S9. Radio-chromatograms of the PBS stability assays at low molar activity. (A) $[^{64}Cu][Cu(DO4S)]^{2+}$, (B) $[^{64}Cu][Cu(DO3S)]^{2+}$, (C) $[^{64}Cu][Cu(DO3SAm)]^{2+}$ and (D) $[^{64}Cu][Cu(DO2A2S)]$.

Table S1. [^{64}Cu]Cu^{2+} incorporation in the cyclen-based ligands and NODAGA-RGD at several molar activities (pH 4.5)

	Molar Activity [MBq/nmol]	Radiochemical Incorporation [%]			
		NODAGA-RDG	DO2A2S	DO4S	DO3S
RT	500	1 ± 4	2 ± 1	0	0
	250	1 ± 3	2 ± 3	0	0
	100	3 ± 2	14 ± 4	0	0
	50	24 ± 6	27 ± 6	0	0
	25	58 ± 11	54 ± 9	0	-
	10	99 ± 13	82 ± 14	38 ± 11	-
	5	99 ± 1	95 ± 1	75 ± 5	22 ± 3
	3	99.0 ± 0.3	98 ± 2	82 ± 5	47 ± 5
	1	99 ± 1	97 ± 4	100	57 ± 1
	0.01	100	100	100	98 ± 2
	**Molar Activity [MBq/nmol]**	**Radiochemical Incorporation [%]**			
		**NODAGA-RDG**	**DO2A2S**	**DO4S**	**DO3S**
90°C	500	1.0 ± 0.2	9 ± 3	0	0
	250	2.0 ± 0.4	15 ± 4	6 ± 1	0
	100	9 ± 3	57 ± 19	28 ± 5	0
	50	79 ± 10	93 ± 11	82 ± 3	-
	25	93 ± 5	99.0 ± 0.3	90 ± 0.1	8 ± 7
	10	99.0 ± 0.2	99.0 ± 0.1	99.9 ± 0.1	-
	5	99.0 ± 0.9	99.0 ± 0.6	99.9 ± 0.1	82 ± 3
	3	99.0 ± 0.1	99.0± 0.5	99.9 ± 0.1	-
	1	99.0 ± 0.1	99.0 ± 0.2	99.9 ± 0.1	100

Table S2. [^{64}Cu]Cu^{2+} incorporation in the cyclen-based ligands and NODAGA-RGD at several molar activities (pH 7.0)

	Molar Activity [MBq/nmol]	Radiochemical Incorporation [%]		
		NODAGA-RDG	DO2A2S	DO4S
RT	500	1 ± 3	9 ± 2	0
	250	1 ± 1	19 ± 1	0 ± 0.5
	100	0.6 ± 0.2	52 ± 8	3 ± 2
	50	0.6 ± 6	92 ± 6	25 ± 16
	25	50 ± 9	99.4 ± 0.6	86 ± 14
	10	97.1 ± 0.1	98.7 ± 0.2	99.9 ± 0.1
	5	98.2 ± 0.1	98.3 ± 0.1	99.9 ± 0.1
	3	98.2 ± 0.1	98.7 ± 0.1	99.9 ± 0.1
	1	97.9 ± 0.1	98.4 ± 0.1	99.9 ± 0.1
	**Molar Activity [MBq/nmol]**	**Radiochemical Incorporation [%]**		
		**NODAGA-RDG**	**DO2A2S**	**DO4S**
90°C	500	2.4 ± 0.1	2.4 ± 0.1	0
	250	1.7 ± 0.1	4.1 ± 0.1	4.7 ± 0.1
	100	1.1 ± 1	68 ± 9	11 ± 3
	50	22 ± 19	95 ± 2	39 ± 8
	25	83 ± 16	99 ± 8	83 ± 17
	10	98.5 ± 0.1	99.5 ± 0.1	99.9 ± 0.1
	5	98.5 ± 0.1	99.4 ± 0.1	99.9 ± 0.1
	2	98.4 ± 0.1	99.2 ± 0.1	99.9 ± 0.1
	1	98.4 ± 0.1	99.5 ± 0.1	99.9 ± 0.1

Table S3. [^{64}Cu]Cu^{2+} incorporation in the non-cyclen-based ligands and NODAGA-RGD at several molar activities (pH 7.0)

	Molar Activity [MBq/nmol]	Radiochemical Incorporation [%]		
		NODAGA-RDG	TRI4S	TE4S
RT	500	1 ± 3	0	0
	250	1 ± 1	0	0
	100	0.6 ± 0.2	32 ± 4	0
	50	0.6 ± 6	57 ± 3	23 ± 2
	25	50 ± 9	76 ± 3	24 ± 5
	10	97.1 ± 0.1	99.9 ± 0.1	32 ± 3
	5	98.2 ± 0.1	99.9 ± 0.1	41.5 ± 3
	3	98.2 ± 0.1	99.9 ± 0.1	40 ± 4
	1	97.9 ± 0.1	99.9 ± 0.1	39 ± 4
